# Comparison of the Cumulative Efficacy and Safety of Chloroquine, Artesunate, and Chloroquine-Primaquine in *Plasmodium vivax* Malaria

**DOI:** 10.1093/cid/ciy319

**Published:** 2018-06-08

**Authors:** Cindy S Chu, Aung Pyae Phyo, Khin Maung Lwin, Htun Htun Win, Thida San, Aye Aye Aung, Rattanaporn Raksapraidee, Verena I Carrara, Germana Bancone, James Watson, Kerryn A Moore, Jacher Wiladphaingern, Stéphane Proux, Kanlaya Sriprawat, Markus Winterberg, Phaik Yeong Cheah, Amy L Chue, Joel Tarning, Mallika Imwong, François Nosten, Nicholas J White

**Affiliations:** 1 Department of Medicine, Shoklo Malaria Research Unit, Mahidol–Oxford Tropical Medicine Research Unit, Faculty of Tropical Medicine, Mahidol University, Mae Sot, Thailand; 2 Centre for Tropical Medicine and Global Health, Nuffield Department of Medicine, University of Oxford, United Kingdom; 3 Department of Haematology, Shoklo Malaria Research Unit, Mahidol–Oxford Tropical Medicine Research Unit, Faculty of Tropical Medicine, Mahidol University, Mae Sot; 4 Clinical Therapeutics Unit, Mahidol–Oxford Tropical Medicine Research Unit, Faculty of Tropical Medicine, Mahidol University, Bangkok, Thailand; 5 Department of Statistics, Macfarlane Burnet Institute for Medical Research and Public Health; 6 Centre for Epidemiology and Biostatistics, Melbourne School of Population and Global Health, University of Melbourne, Victoria, Australia; 7 Department of Data Management, Shoklo Malaria Research Unit, Mahidol–Oxford Tropical Medicine Research Unit, Faculty of Tropical Medicine, Mahidol University, Mae Sot; 8 Department of Microscopy, Shoklo Malaria Research Unit, Mahidol–Oxford Tropical Medicine Research Unit, Faculty of Tropical Medicine, Mahidol University, Mae Sot; 9 Malaria Laboratory, Shoklo Malaria Research Unit, Mahidol–Oxford Tropical Medicine Research Unit, Faculty of Tropical Medicine, Mahidol University, Mae Sot; 10 Department of Clinical Pharmacology, Mahidol–Oxford Tropical Medicine Research Unit; 11 Department of Bioethics and Engagement, Mahidol–Oxford Tropical Medicine Research Unit; 12 Department of Molecular Tropical Medicine and Genetics, Faculty of Tropical Medicine, Mahidol University, Bangkok, Thailand

**Keywords:** *Plasmodium vivax*, radical cure, relapse, chloroquine, primaquine

## Abstract

**Background:**

Chloroquine has been recommended for *Plasmodium vivax* infections for >60 years, but resistance is increasing. To guide future therapies, the cumulative benefits of using slowly eliminated (chloroquine) vs rapidly eliminated (artesunate) antimalarials, and the risks and benefits of adding radical cure (primaquine) were assessed in a 3-way randomized comparison conducted on the Thailand-Myanmar border.

**Methods:**

Patients with uncomplicated *P. vivax* malaria were given artesunate (2 mg/kg/day for 5 days), chloroquine (25 mg base/kg over 3 days), or chloroquine-primaquine (0.5 mg/kg/day for 14 days) and were followed for 1 year. Recurrence rates and their effects on anemia were compared.

**Results:**

Between May 2010 and October 2012, 644 patients were enrolled. Artesunate cleared parasitemia significantly faster than chloroquine. Day 28 recurrence rates were 50% with artesunate (112/224), 8% with chloroquine (18/222; *P* < .001), and 0.5% with chloroquine-primaquine (1/198; *P* < .001). Median times to first recurrence were 28 days (interquartile range [IQR], 21–42) with artesunate, 49 days (IQR, 35–74) with chloroquine, and 195 days (IQR, 82–281) with chloroquine-primaquine. Recurrence by day 28, was associated with a mean absolute reduction in hematocrit of 1% (95% confidence interval [CI], .3%–2.0%; *P* = .009). Primaquine radical cure reduced the total recurrences by 92.4%. One-year recurrence rates were 4.51 (95% CI, 4.19–4.85) per person-year with artesunate, 3.45 (95% CI, 3.18–3.75) with chloroquine (*P* = .002), and 0.26 (95% CI, .19–.36) with chloroquine-primaquine (*P* < .001).

**Conclusions:**

Vivax malaria relapses are predominantly delayed by chloroquine but prevented by primaquine.

**Clinical Trials Registration:**

NCT01074905.

## Teaser

Vivax malaria relapses frequently even in low-transmission settings. Chloroquine delays but does not prevent recurrences. Adding primaquine to a slowly eliminated schizonticide significantly reduces recurrences and improves hematocrit, but this advantage is offset by hemolysis in G6PD-deficient females.

*Plasmodium vivax*, which is becoming the main cause of malaria outside sub-Saharan Africa, exerts considerable morbidity by causing repeated relapses. In Southeast Asia and Oceania, *P. vivax* relapses early and frequently [1, 2]. Prevention of relapse requires radical treatment with primaquine (PMQ), which carries the attendant risk of hemolysis in glucose-6-phosphate dehydrogenase (G6PD)–deficient persons. As G6PD deficiency is common in tropical regions and testing is seldom available, radical-cure PMQ regimens are often not given despite being widely recommended. For nearly 70 years the standard treatment for *P. vivax* malaria has been chloroquine (CQ), although resistance is increasing in many areas [3]. Chloroquine is eliminated slowly, resulting in posttreatment suppression of emerging *P. vivax* malaria recurrences for over a month following treatment. The artemisinin derivatives are more potent [4] but are rapidly eliminated and therefore do not provide posttreatment suppression of recurrent infections. An important question is whether slowly eliminated antimalarials delay or prevent early relapses [5]. Killing all asexual parasites emerging from the liver 2 weeks after the acute illness, would reduce the total number of relapses substantially and would be of public health benefit. This would support use of slowly eliminated partner antimalarial drugs if artemisinin combination therapies are used to treat *P. vivax* malaria. To answer this question and to assess the radical curative efficacy of PMQ (in a context where it was not routinely given for radical cure), a 3-way comparison was conducted of CQ, artesunate (AS), and CQ + PMQ.

## METHODS

Shoklo Malaria Research Unit (SMRU) is located along the border of northwest Thailand. Malaria transmission in this tropical mountainous region is low and seasonal [6, 7]. All patients ≥6 months and weighing ≥7 kg presenting to SMRU outpatient clinics with microscopy-confirmed uncomplicated *P. vivax* monoinfections were screened. Exclusion criteria included pregnancy, severe malaria, hematocrit <25%, allergies to antimalarial drugs, blood transfusion in the last 3 months, antimalarial use in the last 4 weeks, and inability to comply with study procedures. Written informed consent was obtained from patients or their carers with a literate witness present if unable to read in their preferred language.

### Procedures

A full medical history and physical examination were performed. A malaria smear, hematocrit, complete blood count, G6PD fluorescent spot test, and urine β-human chorionic gonadotropin pregnancy test were performed and blood samples taken for human G6PD, parasite genotyping, and parasite culture (for parasitemia >300/500 white blood cells with >80% ring stages [8]). All enrollment malaria blood smears were recounted at the central laboratory; parasite density was the mean of the 2 readings. All follow-up positive slides and 10% of randomly selected negative slides were rechecked. G6PD genotyping for Mahidol variant was performed on all female participants using an established polymerase chain reaction–restriction fragment length polymorphism protocol [9]. Capillary whole blood CQ and desethylchloroquine concentrations were measured using a liquid chromatography–mass spectrometry assay (Supplementary Appendix 1).

Randomization was computer generated in blocks of 12 and assigned by phone call to a staff member who was independent from study activities. Subjects were randomized (unblinded) to either: (1) AS 2 mg/kg/day for 5 days (Medopharm, India and/or Guilin Pharmaceuticals, China; 50-mg tablets); (2) CQ 25 mg base/kg divided over 3 days (10 mg/kg, 10 mg/kg, 5 mg/kg) (Maneesh Pharmaceuticals, Ltd, India and Medopharm, India; 250-mg tablets); or (3) CQ given concomitantly with PMQ 0.5 mg base/kg/day (given within 15 minutes after food) for 14 days (Maneesh Pharmaceuticals, Ltd., India and Government Pharmaceutical Organization, Thailand; 15-mg tablets).

Patients with G6PD deficiency were randomized to arms 1 or 2 only. All doses were supervised. For children unable to swallow tablets, whole tablets were crushed and mixed with water, and the correct dose was administered in suspension. The full dose was repeated for vomiting within 30 minutes. Half the dose was repeated for vomiting between 30 and 60 minutes.

### Follow-up

Temperature and a malaria blood smear were measured daily until afebrile and parasite negative. Fever clearance was the interval to an aural temperature <37.5°C on 2 consecutive measurements. During follow-up visits, a medical history, physical examination, malaria smear, hematocrit, adverse event record, concomitant medication review, and eligibility assessment were performed. Chloroquine levels were measured on day 6 (±1 day) and at recurrences within 28 days of treatment. Recurrences of *P. vivax* malaria were confirmed by microscopy, enrollment procedures repeated, and patients re-treated with the same study drug. Follow-up visits and procedures were restarted as if newly recruited. The total follow-up duration remained 1 year from enrollment. If *Plasmodium falciparum* or mixed infection malaria occurred, standard AS-mefloquine treatment was given and follow-up continued without interruption. Radical cure with PMQ was given after 9 recurrences or earlier if clinically indicated. Subjects were censored if consent was withdrawn or lost to follow-up. Adverse events were recorded daily during treatment and weekly until day 28. Hematinics were given if the hematocrit fell below 30% (<34% in children <2 years old) and a blood transfusion if it fell below 18%. If cyanosis was evident, transcutaneous methemoglobin levels were checked (Masimo Radical-7).

### Sample Size Calculation

An estimated 10% of CQ-treated patients were expected to have *P. vivax* recurrences within 28 days. A sample size of 200 patients per treatment arm allowed a difference of >8% to be detected using the log-rank test with 80% power, 95% confidence, and 20% loss to follow-up. To account for exclusion of patients with G6PD deficiency, an additional 60 patients were added for a final sample size of 660.

### Statistical Analyses

The primary endpoint was *P. vivax* malaria recurrence within 28 days. Secondary endpoints were interval to first recurrence, *P. vivax* malaria recurrences during 1 year, relationships with CQ levels, and adverse events within 28 days. All evaluable subjects were included in a modified intention-to-treat analysis to assess antimalarial drug efficacy. Associations were assessed using χ^2^ or Fisher exact tests and group comparisons and relationships assessed were using Student *t* test, linear regression, nonparametric K-sample test, Wilcoxon rank-sum test, or logistic regression as appropriate. The multivariate analysis included all variables significant at the 5% level in a univariate model. Steady-state hematocrit was defined as the mean of all measurements during the year after day 42 provided there was no interim malaria recurrence [10]. Efficacy was analyzed using the Kaplan-Meier method with Cox regression used to evaluate the risk factors for recurrence (ie, sex, age, hematocrit, or parasitemia) and the effects of CQ levels. An Anderson-Gill extended Cox model [11] was fitted to estimate the total effect (effect of the treatment + effect of dependency between recurrences) of treatment on the hazard of recurrence. The duration of posttreatment prophylaxis after CQ (difference in median times to recurrent infection between the CQ and AS groups) was estimated as 20 ± 5 days. Confidence intervals (CIs) were calculated by bootstrapping. Statistical analyses were performed using Stata version 13 and 14 software (StataCorp, College Station, Texas).

This study was approved by the Mahidol University Faculty of Tropical Medicine Ethics Committee (MUTM 2010-006) and the Oxford Tropical Research Ethics Committee (OXTREC 04-10), and was registered at ClinicalTrials.gov (NCT01074905).

## RESULTS

### Patient Characteristics

Between May 2010 and October 2012, 925 patients with acute *P. vivax* malaria were screened; 600 G6PD-normal and 55 G6PD-deficient patients were enrolled (Supplementary Figure 1). Patient demographics and clinical characteristics were similar between groups (Supplementary Tables 1 and 2) although more males (293/429 [68%]) than females (107/215 [50%]) worked in the forest or on farms where malaria transmission is higher (*P* < .001). Previous malaria (total 737 episodes) was reported in 354 of the 644 (55%) evaluable patients. Children <5 years old were more likely to have hepatomegaly (22%; *P* = .001) and splenomegaly (19%; *P* < .001) than adults (Supplementary Table 3). The median (interquartile range [IQR]) follow-up durations were similar between all groups: AS (314 [166–365] days), CQ (364 [157–365] days), and CQ + PMQ (364 [197–365] days) (*P* = .533). Overall, 281 of 644 (44%) patients did not complete the full 1-year study, of whom 240 of 281 (85%) were lost to follow-up. Males (203 of 429 [47%]) were more likely than females (78 of 215 [36%]) to discontinue follow-up (*P* = .008). Adults (207 of 402 [52%]) were more likely to discontinue than children (73/241 [30%]) (*P* > .001).

### Treatment Efficacy

By day 1, 81% of patients in the AS group defervesced compared with 67% in the CQ group (*P* = .035) and 60% in the CQ + PMQ group (*P* = .002). More than 95% of patients were afebrile by day 2. More patients also cleared parasitemia by day 1 following AS (70%) compared to CQ (22%; *P* < .001) and CQ + PMQ (28%; *P* < .001) (Supplementary Table 4). Day 28 cure rates (95% CI) were 50% (43.3%–56.7%) for AS, 91.9% (87.5%–95.1%) for CQ, and 99.5% (97.2%–100%) for CQ + PMQ (*P* < .001; Table 1).

**Table 1.** Description of First *Plasmodium vivax* Recurrence

Treatment Arm	No.	First Recurrence by 1 y, No. (%; 95% CI)	Median Time to First Recurrence, d^a^, (IQR; Range)	Recurrence by Day 28, No. (%)	Cumulative Risk of Recurrence by Day 28^b^, OR (95% CI)	*P* Value
AS	224	177 (79; 73–84)	28 (21–42; 7–252)	112 (50)	Comparator	
CQ	222	165 (74; 68–80)	49 (35–74; 13–354)	18 (8.1)	0.088 (.051–.153)	<.001
CQ + PMQ	198	35 (18; 13–24)	195 (82–281; 13–364)	1 (0.5)	0.005 (.001–.037)	<.001

Abbreviations: AS, artesunate; CI, confidence interval; CQ, chloroquine; IQR, interquartile range; OR, odds ratio; PMQ, primaquine.

^a^Nonparametric K-sample test was used to compare medians between groups; all *P* values < .001.

^b^Logistic regression was used to compare the difference between groups.

### First Recurrences

**Figure 1.**
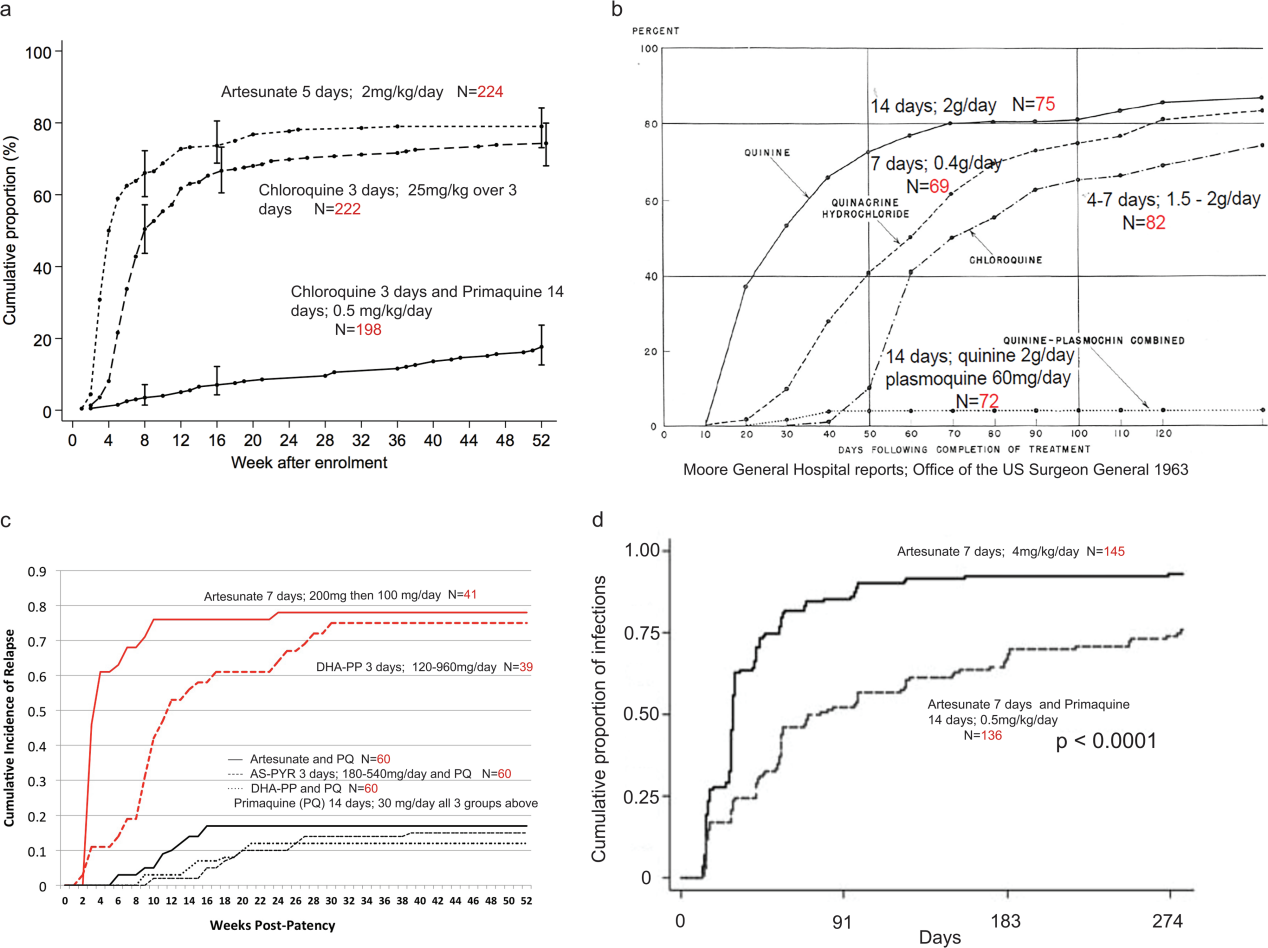
Cumulative proportions of first recurrences after treatment of the initial *Plasmodium vivax* infection in the current study (*A*) compared with historical data from US soldiers returning from the Pacific [14] (*B*), Indonesian soldiers returning from Papua, Indonesia [15, 16] (*C*), and children aged 1–5 years living in East Sepik Province, Papua New Guinea [17] (*D*). Note that the cumulative recurrence curve of more slowly eliminated schizonticides (ie, chloroquine, dihydroartemisinin-piperaquine) is shifted to the right, compared with rapidly eliminated schizonticides (ie, artesunate). Permission to use (*C*) was obtained from the last author and the figure is also under Creative Commons CC-BY license. Permission to use (*D*) was obtained by Oxford University Press, licence number 4241781058637. Abbreviations: AS, artesunate; AS-PYR, artesunate-pyronaridine; DHA-PP, dihydroartemisinin-piperaquine; PMQ, primaquine.

Overall, recurrences of *P. vivax* parasitemia (n = 1349) occurred in 377 (59%) patients, peaking after the transmission season ended (Supplementary Figure 2). Median intervals to first recurrence were 28 days in the AS group, 49 days in the CQ group, and 195 days in the CQ + PMQ group (*P* < .001; Table 1). Adding PMQ to CQ reduced the *P. vivax* first recurrence rate (n = 377) over 1 year by 85.6%; annual rates per person-year (95% CI) were 1.85 (1.60–2.14) for AS, 1.60 (1.37–1.86) for CQ, and 0.23 (.17–.32) for CQ + PMQ (Table 1 and Figure 1). A history of previous malaria was more common in patients with recurrences (229/377 [61%]) than without recurrence (148/377 [39%]) (*P* < .001). Age and gender did not affect either the risk or the number of *P. vivax* malaria recurrences. The day 6 mean (95% CI) capillary whole blood CQ drug concentrations in those with and without a first recurrence were 353 (330–377) ng/mL and 397 (358–435) ng/mL, respectively (*P* = .061). Patients with first recurrences between day 28 and 42 (n = 66) had lower day 6 CQ levels (330 [296–365] ng/mL) compared to patients with no recurrence (379 [355–404] ng/mL) (*P* = .028). Of the 47 patients who had levels measured at the time of this recurrence, all were <100 ng/mL. In the 18 patients who had a first recurrence before day 28, day 6 levels were similar to those without early recurrences (difference in CQ level, 21 [95% CI –53.8 to 95.6] ng/mL; *P* = .582), and 4 (22%) had levels ≥100 ng/mL on the day of recurrence. Thus, only 4 of 216 infections (1.9% [95% CI, .1%–3.7%]) were CQ resistant. In vitro susceptibility assessments were successful in 95 cases; geometric mean CQ 50% inhibitory concentration (IC_50_) was 19.7 (95% CI, 16.6–23.4) ng/mL. Seven isolates (7.4%) had an IC_50_ >50 ng/mL, suggesting resistance. Chloroquine susceptibilities were not available for the 4 patients with early (<28 days) recurrences following CQ.

### Second and Subsequent Recurrences

**Figure 2.**
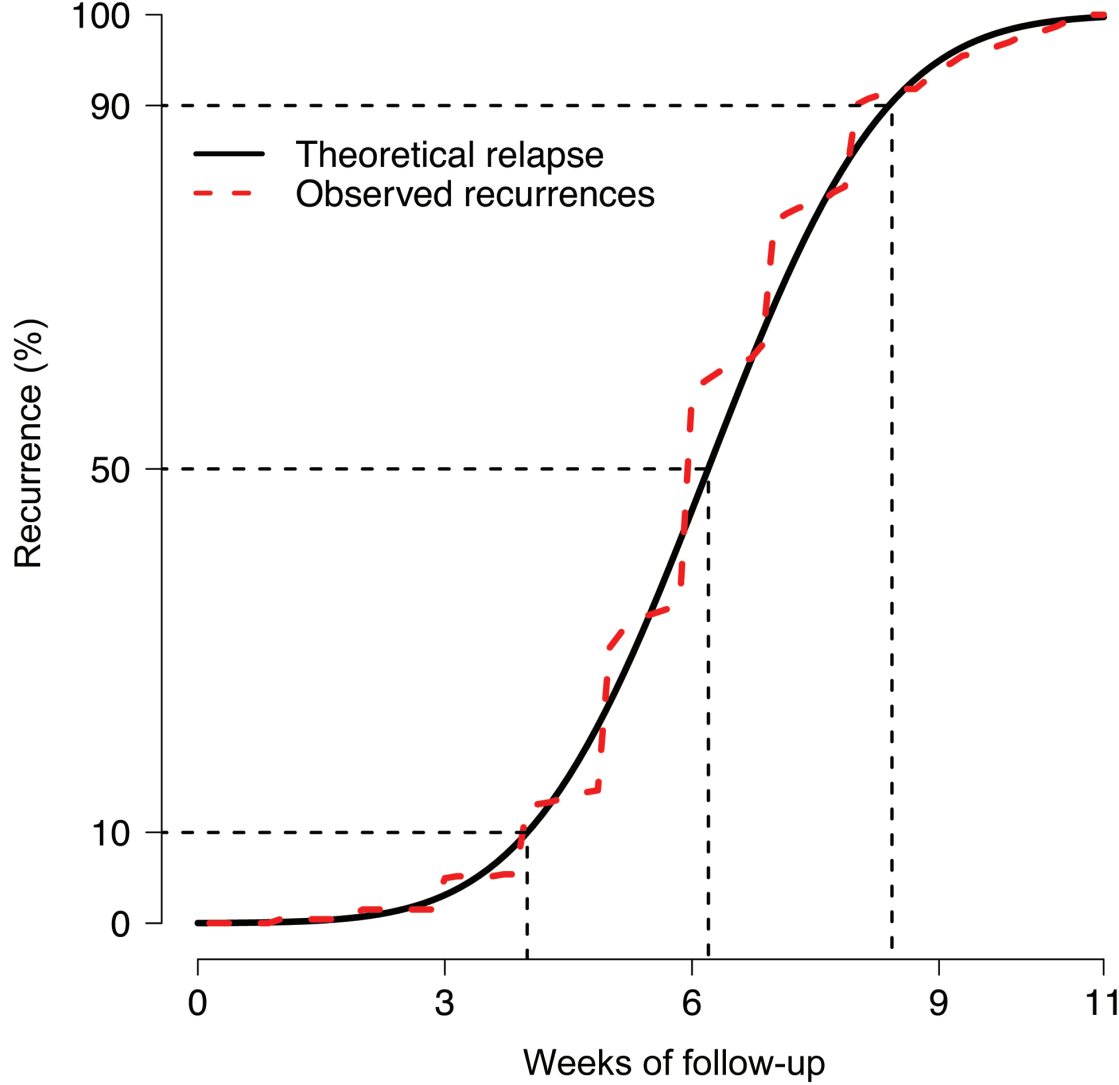
Modeling the proportion of relapses occurring after monotherapy with chloroquine (CQ). If the background rate of new *Plasmodium vivax* infection is taken into account (estimated from the CQ + primaquine group), an estimated 90% of all relapses in the CQ group occur within 9 weeks.

The proportions (95% CI) of patients who had a second *P. vivax* recurrence were similar in the AS (79% [72%–84%]) and CQ (78% [71%–84%]) groups, but were substantially lower in the CQ + PMQ group (14% [5%–30%]) (*P* < .001). The corresponding proportions with third recurrences were as follows: AS, 76% (68%–83%) and CQ, 71% (63%–79%) vs none in the CQ + PMQ group (*P* < .001). Over the 1-year follow-up, there were more recurrences per person-year in the AS (4.51) than CQ group (3.45; hazard ratio [HR] vs AS, 0.77; *P* = .002), and substantially fewer in the CQ + PMQ group (0.26; HR vs AS, 0.06; *P* < .001) (Table 2). When the posttreatment prophylactic effect of CQ was taken into account, the adjusted mean recurrence rate following CQ was 4.30 (95% CI, 3.8–4.8) per person-year, only 5% less than with AS. This suggests that CQ delays rather than prevents relapses (Supplementary Figure 3). If the background rate of new infections (estimated from the CQ + PMQ group) is subtracted from the recurrence rate, then the estimated relapse rate per person-year was 4.25 in the AS group and 3.19 in the CQ group, with 90% of all relapses in the CQ group occurring within 9 weeks (Figure 2). Independent risk factors for repeated recurrences in the AS and CQ groups were being male (HR, 1.27 [95% CI, 1.05–1.54]; *P* = .013) and having a previous history of malaria (HR, 1.27 [95% CI, 1.06–1.51]; *P* = .008). There were no significant risk factors for repeated recurrence in the CQ + PMQ group. Over all the recurrences (n = 1349), compared with CQ alone, adding PMQ radical cure reduced recurrences by 92.4%.

**Table 2.** *Plasmodium vivax* Recurrences Within 1 Year of Follow-up

Treatment Arm	No.	Total Recurrences in 1 y, No. (%)	Median No. of Recurrences (Range)	Total Effect HR (95% CI)	*P* Value	Follow-up Time, y	Rate of Recurrence/PY (95% CI)
AS	224	722 (54)	2 (0–14)	Comparator		159	4.51 (4.19–4.85)
CQ	222	587 (43)	2 (0–13)	0.77 (.65–.91)	.002	167	3.45 (3.18–3.75)
CQ + PMQ	198	40 (3)	0 (0–2)	0.06 (.04–.08)	<.001	154	0.26^a^ (.19–.36)

Abbreviations: AS, artesunate; CI, confidence interval; CQ, chloroquine; HR, hazard ratio; PMQ, primaquine; PY, person-years.

^a^Rate of relapse: rate of recurrence minus rate of recurrence in the CQ + PMQ arm (under the assumption that all recurrences in the CQ-PMQ group are new infections, and that the risk of infection is equivalent across treatment groups).

The mean day 6 CQ drug level in recurrent infections (384 [95% CI, 370–399] ng/mL) was similar to the first treatment (397 ng/mL; *P* = .584), but in recurrent infections lower levels were neither associated with risk nor timing of subsequent recurrence. Chloroquine levels exceeded 100 ng/mL in 10 of 225 (4%) infections on the day of subsequent recurrence. Of the 58 patients with a subsequent recurrence before day 28, 16 (28%) had levels >100 ng/mL.

### Hematological Consequences

**Figure 3.**
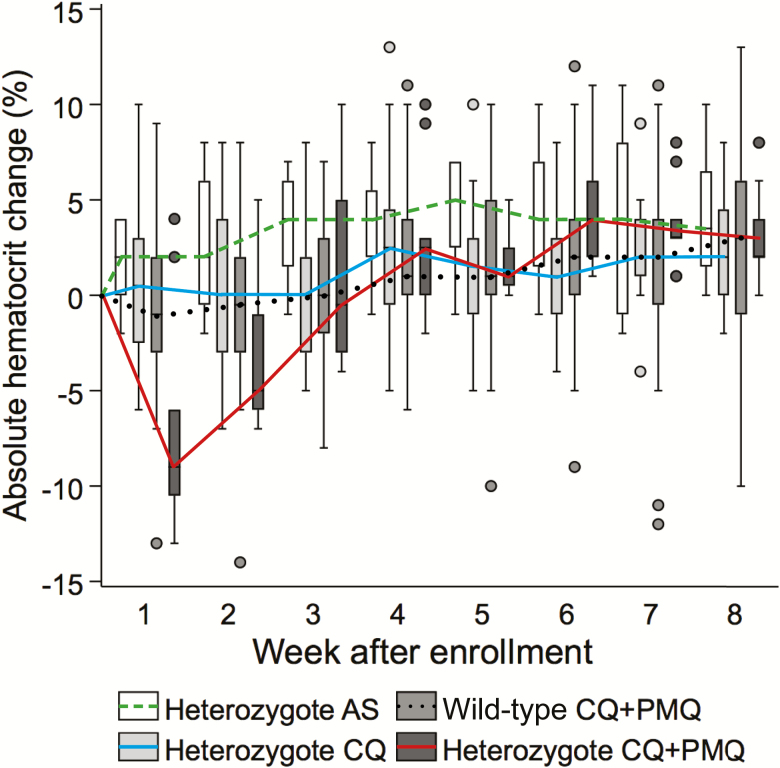
Median absolute hematocrit changes in glucose-6-phosphate dehydrogenase (G6PD)–heterozygous female patients from enrollment to week 8 of the acute *P. vivax* infection. Patients who had recurrences during the first 8 weeks are included. G6PD-homozygous females are not included. Wild-type (G6PD genotype normal) females in the chloroquine (CQ) + primaquine (PMQ) group are shown as a reference. The dots represent outlier values. The number of heterozygotes in each group are as follows: artesunate (AS), n = 8; CQ, n = 16; and CQ + PMQ, n = 10.

The mean (95% CI) acute hematocrit reduction (day 0 to nadir at day 6) was similar in the CQ + PMQ (–0.8% [–1.4% to –0.3%]), AS (–0.8% [–1.2% to –.3%]), and CQ groups (–0.3% [–.8% to .2%]; *P* = .174). In the 43 patients who were anemic at enrollment (hematocrit ≤30%), the mean hematocrit actually increased by day 6 (Supplementary Figure 4). The mean (95% CI) hematocrit recovery (ie, day 6 to steady state) was greater in the CQ + PMQ group (3.8% [3.3%–4.3%]) compared with the AS (3.1% [2.6%–3.6%]; *P* = .035) and CQ groups (2.7% [2.2%–3.1%]; *P* = .001). Overall, the median fractional hematocrit recovery (day 6 to steady state) was 7.4% (IQR, 2.8%–1.3%) (Supplementary Figure 5). Recurrences by week 4 were associated with a 1% absolute mean hematocrit reduction (95% CI, .3–2) compared to the remainder (*P* = .009) (Supplementary Figure 6). By week 20 (~5 months), the mean hematocrits of all groups had converged to the study population mean irrespective of interim recurrences. Independent risk factors associated with greater acute hematocrit reductions were being G6PD heterozygote in the CQ + PMQ group (mean absolute hematocrit fall of –6.2% [95% CI, –8.4% to –3.9%]; *P* < .001), age >15 years (–1.0% [95% CI, –2.0% to –.01%]; *P* = .048), and having a previous history of malaria (–0.66% [95% CI, –1.2% to –0.1%]) (*P* = .021; Figure 3). Over all the recurrences, the maximum acute hematocrit reduction was >10% in only 9 patients (1.4%), all of whom had pretreatment values >40%. Mean acute hematocrit reductions and recoveries over all recurrences were similar among the 3 treatment groups (Supplementary Table 5).

### Adverse Events up to Day 28

Adverse event rates were similar in all treatment groups. Abdominal pain was more common in the CQ + PMQ than the CQ group but not the AS group (Table 3). More patients in the CQ + PMQ group were treated for anemia than in the AS or CQ groups; 4 of 9 (44%) were G6PD-heterozygous females (Table 3). One death from accidental head injury occurred 18 days after completing AS treatment. No blood transfusions were required.

**Table 3.** Comparison of Adverse Events Between Treatment Arms

Adverse Event	No. (%)	RR Ratio (95% CI)	*P* Value^b^
Abdominal pain			
AS	10/207 (5)	0.52 (.20–1.37)	.184
CQ	5/223 (2)	0.24 (.08–.74)	.013
CQ + PMQ	8/90 (9)	Comparator	NA
Treatment for anemia^a^			
AS	4/207 (2)	0.18 (.05–.59)	.005
CQ	7/223 (3)	0.29 (.11–.81)	.018
CQ + PMQ	9/90 (10)	Comparator	NA

Abbreviations: AS, artesunate; CI, confidence interval; CQ, chloroquine; NA, not applicable; PMQ, primaquine; RR, relative risk.

^a^Anemia was defined as a hematocrit <30% (<33% if <2 years old).

^b^Logistic regression was used to compare differences between groups.

## DISCUSSION

Although there is in vivo and in vitro evidence of CQ resistance on the northwestern border of Thailand [12, 13], most *P. vivax* malaria infections remain sensitive and CQ remains clinically efficacious in treatment. In areas with high-grade CQ resistance in *P. vivax*, national malaria programs now recommend artemisinin combination therapies. The main therapeutic challenge is the high relapse rate. In Southeast Asia and Oceania, *P. vivax* relapses frequently and repeatedly. Following rapidly eliminated drugs such as AS or quinine, relapses become patent approximately 3 weeks after starting treatment. More slowly eliminated drugs such as CQ suppress the asexual parasitemia of the first relapse, but there has been uncertainty whether the first relapse observed after these drugs derives from protracted suppression (ie, delay) of the first relapse, or clearance of this infection and emergence of the second relapse. This study, together with previous data [14–17] (Figure 1), supports the former explanation—that is, relapses are predominantly delayed rather than prevented.

More than 90% of all *P. vivax* recurrences (mainly relapses) occurred by week 16. This suggests that studies evaluating antirelapse treatments in Southeast Asia and Oceania should have at least 16 weeks (~4 months) of follow-up to capture the majority of relapses. Primaquine (total dose 7 mg/kg) was highly efficacious with an estimated antirelapse (radical cure) overall efficacy of at least 92%. Primaquine also has asexual stage activity [18] which would be expected to augment cure rates in resistant infections. Some of the apparent radical cure failures may have been explained by reduced cytochrome P450 2D6 (CYP2D6) PMQ bioactivation [19]. Approximately 20% of the population in the study area carries the CYP2D6*10 allele [20], so 4%–5% of patients would have been intermediate metabolizers. CYP2D6 genotyping results from this study will be reported elsewhere. The cumulative benefit of providing radical cure in preventing *P. vivax* malaria is enormous. In the Southeast Asia region, an estimated 4.6 million cases would have been prevented if all *P. vivax* infections were treated with PMQ radical cure [21].

The main clinical concern both from recurrent *P. vivax* malaria and from PMQ treatment is anemia. In this low-transmission area with ready access to diagnosis and treatment, recurrent *P. vivax* malaria caused only small reductions in hematocrit; these reductions lessened with successive recurrences [22]. In contrast, on the island of New Guinea where malaria transmission is high, vivax malaria is associated with life-threatening anemia in young children. However, using PMQ caused substantially greater hematocrit reductions (~8 times the effect of malaria) in G6PD-heterozygous females [23]. Even so, no blood transfusions were required. Thus, the major benefit from radical cure with PMQ (in the higher doses necessary in SE Asia) in preventing recurrent malaria and attenuating any associated anemia is offset by the risk to G6PD-heterozygous females diagnosed as “G6PD normal” with current tests who may still have clinically significant oxidant hemolysis [24, 25].

One of the study limitations was incomplete follow-up. This resulted from a combination of migration, environmental factors (flooding), the nature of forest work for young males, and long study duration.

In conclusion, CQ continues to be an efficacious treatment of *P. vivax* malaria along the Thailand–Myanmar border, although low-grade resistance has emerged. The benefits of slowly eliminated antimalarials in delaying early relapses are small in comparison with high-dose PMQ radical cure, which prevents nearly all relapses. However, when only G6PD qualitative screening is used, this regimen may cause significant hemolysis in G6PD-deficient heterozygous females. Despite this, PMQ should be used more widely. Greater availability of quantitative G6PD testing or development of safer radical curative regimens would ameliorate the risks.

## Supplementary Data

Supplementary materials are available at *Clinical Infectious Diseases* online. Consisting of data provided by the authors to benefit the reader, the posted materials are not copyedited and are the sole responsibility of the authors, so questions or comments should be addressed to the corresponding author.

Supplement Files

## Notes

***Acknowledgments.*** The authors thank the Shoklo Malaria Research Unit staff (especially Arunrot, Mu Mu Shwe Tun, Yin Mon, Nay Lin Soe, Nathar Hlaing, Thida Zin, Htet Htet Chaw, Nwe Wah Lin, and Say Paw) for their commitment and diligence in running this study; Kathy Burgoine, Kasiha (Tabitha) Pilaseng, and Claudia Turner for their valuable contributions; Rob Commons from the Menzies School of Health Research, Australia, for his scientific discussion on anemia; Sue J. Lee from Mahidol Oxford Tropical Research Unit, Thailand, and Yin Bun Cheung from Duke-NUS Graduate Medical School, Singapore, for their statistical support; Verena Carrara and Christine Luxemburger for their insightful commentary; and the Data Safety and Monitoring Board: Ric Price (Oxford, UK), Kevin Baird (Jakarta, Indonesia), Dennis Shanks (Queensland, Australia), and Kasia Stepniewska (Bangkok, Thailand), for their time and commitment.

***Financial support.*** This work was supported by the Wellcome Trust (grant number 089179/Z/09/Z to N. J. W.).

***Potential conflicts of interest.*** All authors: No reported conflicts. All authors have submitted the ICMJE Form for Disclosure of Potential Conflicts of Interest. Conflicts that the editors consider relevant to the content of the manuscript have been disclosed.
