# Supplementary material for: Comparison of the Cumulative Efficacy and Safety of Chloroquine, Artesunate, and Chloroquine-Primaquine in Plasmodium vivax Malaria
Source: Clin Infect Dis. 2018 Jun 8;67(10):1543–9. doi: 10.1093/cid/ciy319 (PMC6206118; doi:10.1093/cid/ciy319)
Supplement: Supplement Files [file ciy319_suppl_supplement-files.pdf]

Appendix 1. Description of the LC-MS assay used for the measurement of chloroquine and desethylchloroquine concentrations in whole blood.

Capillary blood samples were processed by solid-phase extraction and quantified using mass spectrometry, operated in the positive mode. The method did not show any evidence of significant ion suppression/enhancement. Stable isotope-labelled internal standards were used to compensate for any unexpected matrix and recovery effects. The lower limit of quantification (LLOQ) was 2.56 ng/mL for chloroquine and 3.36 ng/mL for desethylchloroquine. Three replicates of quality control samples at low, middle, and high concentrations were analysed within each batch of clinical samples to ensure precision and accuracy during drug measurements. Total precision (i.e., relative standard deviation [SD]) for all drug measurements was below 5% during drug quantification. For the statistical data analysis, reported LLOQ values were imputed at half of the quantification limit (i.e. LLOQ/2) to reduce bias from data censoring.

Supplementary Table 1. Patient demographics

Patient characteristic	AS	CQ	CQ+PMQ
Total enrolled, No.	224	222	198
Male, No. (%)	159 (71%)	144 (65%)	126 (64%)
Age in years, median (IQR, range)	19 (13-30, 1.5-62)	18 (13-29, 1.5-62)	18 (11-27, 1.8-63)
Patients 0 - 4 years old, No. (%)	20 (9%)	17 (8%)	17 (9%)
Patients 5 - 15 years old, No. (%)	54 (24%)	71 (32%)	62 (31%)
Patients > 15 years old, No. (%)	150 (67%)	134 (60%)	119 (60%)
G6PD heterozygous females, No. (%)	8 (4%)	16 (7%)	9 (5%)
G6PD hemi or homozygous patients, No. (%)	21 (9%)	18 (8%)	0
Burmese ethnicity, No. (%)	73 (33%)	87 (39%)	70 (35%)
Karen ethnicity, No. (%)	144 (64%)	123 (55%)	116 (59%)
Other ethnicity, No. (%)	7 (3%)	12 (6%)	12 (6%)
Years living in area, median (IQR, range)[a]	3 (1.4-7, 0-35)	3 (1-7, 0-20)[1]	4 (1.8-8, 0-32)[1]
Works on farm, No. (%)	130 (58%)	131 (59%)	106 (54%)
Work in forest, No. (%)	16 (7%)	12 (5%)	5 (2%)
Stay in village, No. (%)	65 (29%)	68 (31%)	77 (39%)
Other workplace, No. (%)	13 (6%)	11 (5%)	10 (5%)

Abbreviations: AS, artesunate; CQ, chloroquine; PMQ, primaquine; G6PD, glucose-6-phosphate dehydrogenase; IQR, interquartile range

[a] If subject lived in the area < 1 month, data was entered as 0

Numeric superscript is the number of missing data

Supplementary Table 2. Patient clinical characteristics

Patient characteristic	AS	CQ	CQ+PMQ
Total enrolled, No.	224	222	198
History of malaria Pf or Pv, No. (%)	123 (55%)	126 (57%)[1]	104 (53%)[1]
History of fever, No. (%)	217 (97%)	217 (98%)[2]	194 (98%)[1]
Days of fever, median (IQR, range)	2 (2-3, 0-10)	2 (2-3, 0-7)[2]	2 (2-3, 0-7)[1]
Presence of chills, No. (%)	147 (66%)	170 (77%)[2]	144 (73%)[1]
Presence of headache, No. (%)	188 (84%)	186 (84%)2	164 (83%)[1]
Presence of cough, No. (%)	59 (26%)	58 (26%)[2]	53 (27%)[1]
Presence of difficulty breathing, No. (%)	13 (6%)	10 (5%)[2]	10 (5%)[1]
Presence of abdominal pain, No. (%)	40 (18%)	38 (17%)[2]	29 (15%)[1]
Presence of diarrhea, No. (%)	7 (3%)	1 (0.5%)[2]	6 (3%)[1]
Temperature °C, mean (SD)	37.6 (0.92)	37.7 (0.99)[1]	37.6 (1.0)[1]
Temperature >=37.5 °C, No. (%)	105 (47%)	103 (46%)[1]	82 (41%)[1]
Body Mass Index, median (IQR, range)	19 (17-21, 11-26)	18 (16-20, 12-35)[3]	19 (16-21, 13-33)[3]
Heart rate per minute, median (IQR, range)	84 (78-96, 50-152)[2]	88 (78-100, 54-146)[1]	86 (76-100, 56-140)[2]
Respiratory rate per minute, median (IQR, range)	25 (24-28, 16-58)	26 (22-28, 16-76)[1]	24 (22-28, 16-42)[1]
SBP mmHg, median (IQR, range)[a]	100 (90-110, 80-160)	100 (90-110, 80-150)	100 (90-110, 70-150)
DBP mmHg, median (IQR, range)[a]	60 (60-70, 40-100)	60 (60-70, 40-90)	60 (60-70, 40-90)
Hepatomegaly, No. (%)	19 (9%)	24 (11%)[1]	25 (13%)[1]
Splenomegaly, No. (%)	11 (5%)	10 (5%)[1]	10 (5%)[1]
Parasitemia, geometric mean (95% CI)	4113 (144 to 47,665)	3920 (168 to 44,211)	3524 (136 to 62,298)[1]
Gametocytemia, No. (%)	211 (94%)	212 (96%)	187 (94%)
Field Hematocrit %, mean (SD)	39 (5.8)	38 (5.3)	38 (5.1)
Hemoglobin g/dL, mean (SD)	12.1 (1.7)[3]	11.8 (1.7)[5]	11.8 (1.7)[6]
White blood count 10^3/µL, mean (SD)	6.8 (2.3)[3]	6.7 (2.1)[5]	6.4 (1.8)[6]
Platelet count 10^3/µL, mean (SD)	126 (63)[3]	128 (66)[5]	128 (64)[8]

Abbreviations: Pf: *P. falciparum*, Pv: *P. vivax*, AS, artesunate; CQ, chloroquine; PMQ, primaquine; IQR, interquartile range; SBP, systolic blood pressure; DBP, diastolic blood presure

[a] Blood pressure performed in subjects >12 years old

Numeric superscript is the number of missing data

Supplementary Table 3. Comparison of previous malaria history and hepatosplenomegaly by age

	No. (%)	Relative risk ratio	95% CI	PValue[b]
Frequency of previous episodes of *P. vivax*[a] malaria				
< 5 years	61/68 (90%)	Comparator	Comparator	Comparator
5 - 15 years	180/231 (78%)	0.41	0.18 to 0.94	0.035
> 15 years	335/438 (77%)	0.37	0.17 to 0.84	0.017
Hepatomegaly				
< 5 years	12/54 (22%)	Comparator	Comparator	Comparator
5 - 15 years	25/187 (13%)	0.54	0.25 to 1.16	0.116
> 15 years	31/403 (8%)	0.29	0.14 to 0.61	0.001
Splenomegaly				
< 5 years	10/54 (19%)	Comparator	Comparator	Comparator
5 - 15 years	11/187 (6%)	0.28	0.11 to 0.69	0.006
> 15 years	10/403 (3%)	0.11	0.04 to 0.19	<0.001

[a] The denominator includes history of *P. vivax* and *P. falciparum*

[b] Logistic regression was used to compare differences between groups.

Supplementary Table 4. Fever and parasite clearance times (days)

Treatment arm	Day 1		Day 2		PValue[a]
	No. (%)	95% CI	No. (%)	95% CI	
Fever clearance					
AS (n=109)	88 (81%)	72 to 88	105 (96%)	91 to 99	Comparator
CQ (n=119)	80 (67%)	58 to 76	119 (100%)	NA	0.035
CQ+PMQ (n=103)	62 (60%)	50 to 70	102 (99%)	95 to 100	0.002
Parasite clearance					
AS (n=224)	157 (70%)	64 to 76	216 (96%)	93 to 98	Comparator
CQ (n=219)[3]	49 (22%)	17 to 29	187 (85%)	80 to 90	<0.001
CQ+PMQ (n=194)[4]	55 (28%)	22 to 35	159 (82%)	76 to 87	<0.001

Abbreviations: AS, artesunate; CQ, chloroquine; PMQ, primaquine; NA, not applicable

Enrolment and follow up visits typically occurred in the morning. Patients who did not present with fever who developed a fever within 1 day (approximately 24 hours) of enrolment are included in this table.

[a] Ordered logistic regression was used to compare the proportions of patients on each successive day

Numeric superscript is the number of missing patients

Supplementary Table 5. Mean hematocrit changes during *Plasmodium vivax* recurrences

Treatment arm	No.	Mean acute absolute hematocrit reduction (95% CI)	PValue[a]	Mean absolute hematocrit recovery (95% CI)	PValue[a]
AS	177	-0.4 (-0.6 to -0.2)	0.874	1.9% (1.6 to 2.1)	0.681
CQ	165	0.1% (-0.2 to 0.3)	0.42	0.8% (0.6 to 1.1)	0.077
CQ+PMQ	35	-0.6% (-1.9 to 0.6)	Comparator	2.2% (0.9 to 3.4)	Comparator

[a] Linear regression was used to compare mean differences between groups

Supplementary Figure 1. Trial diagram

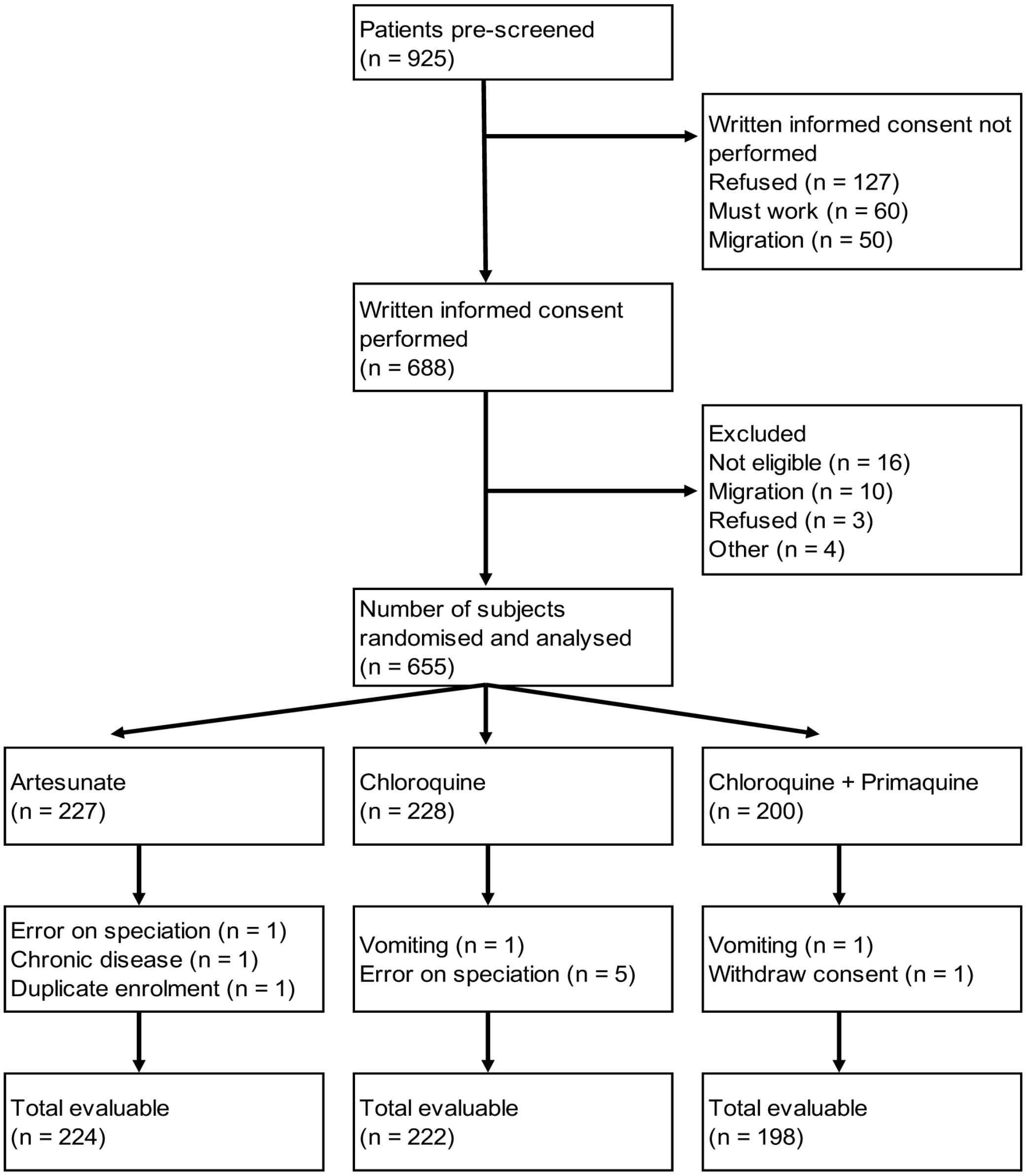

Supplementary Figure 2. Hazard rate of *Plasmodium vivax* recurrences by month

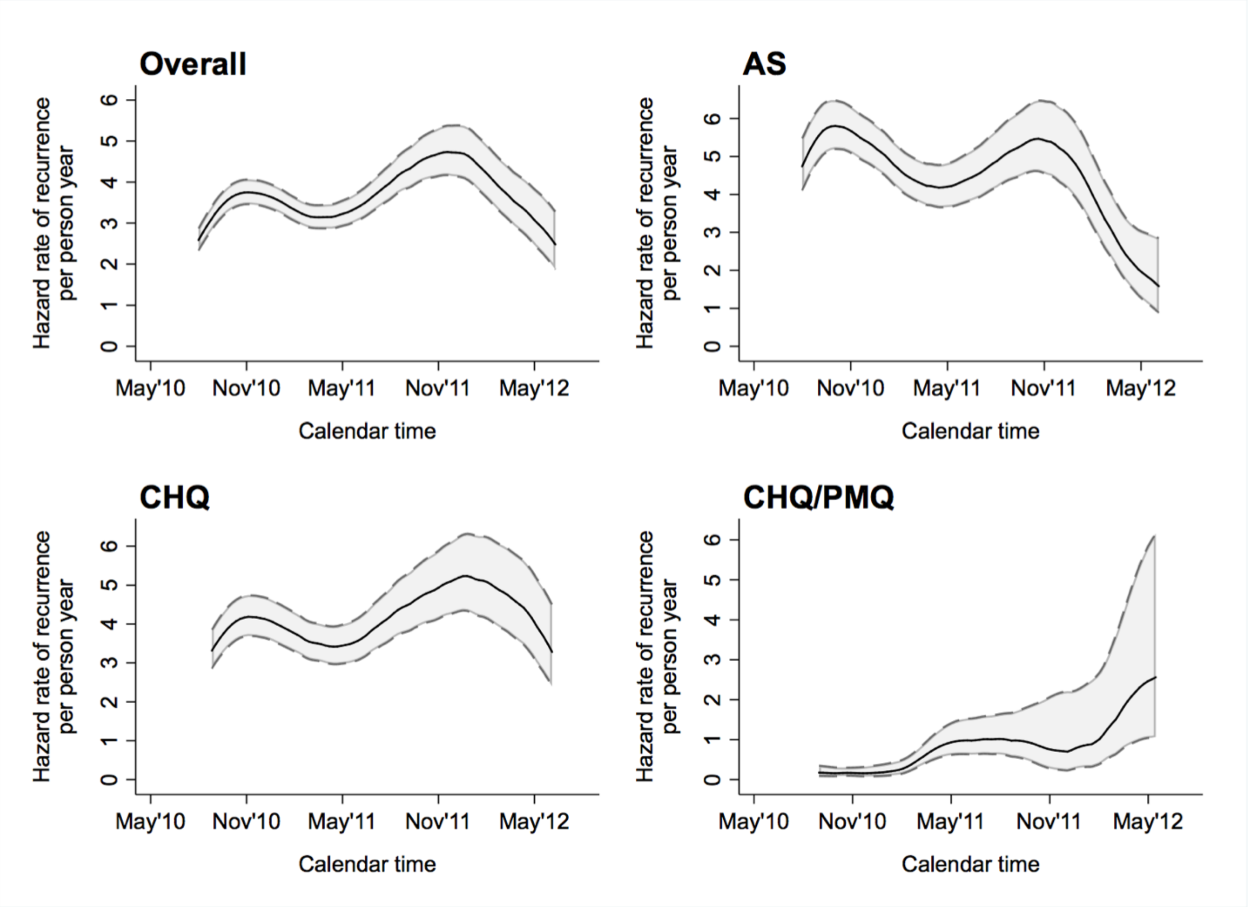

Plasmodium vivax transmission occurs throughout the year. The main transmission season is from June to August. The shaded areas represent 95% CI. Abbreviations: AS, artesunate; CQ, chloroquine; PMQ, primaquine

Supplementary Figure 3. Recurrence rate per person year adjusted for the post-treatment prophylactic period of chloroquine

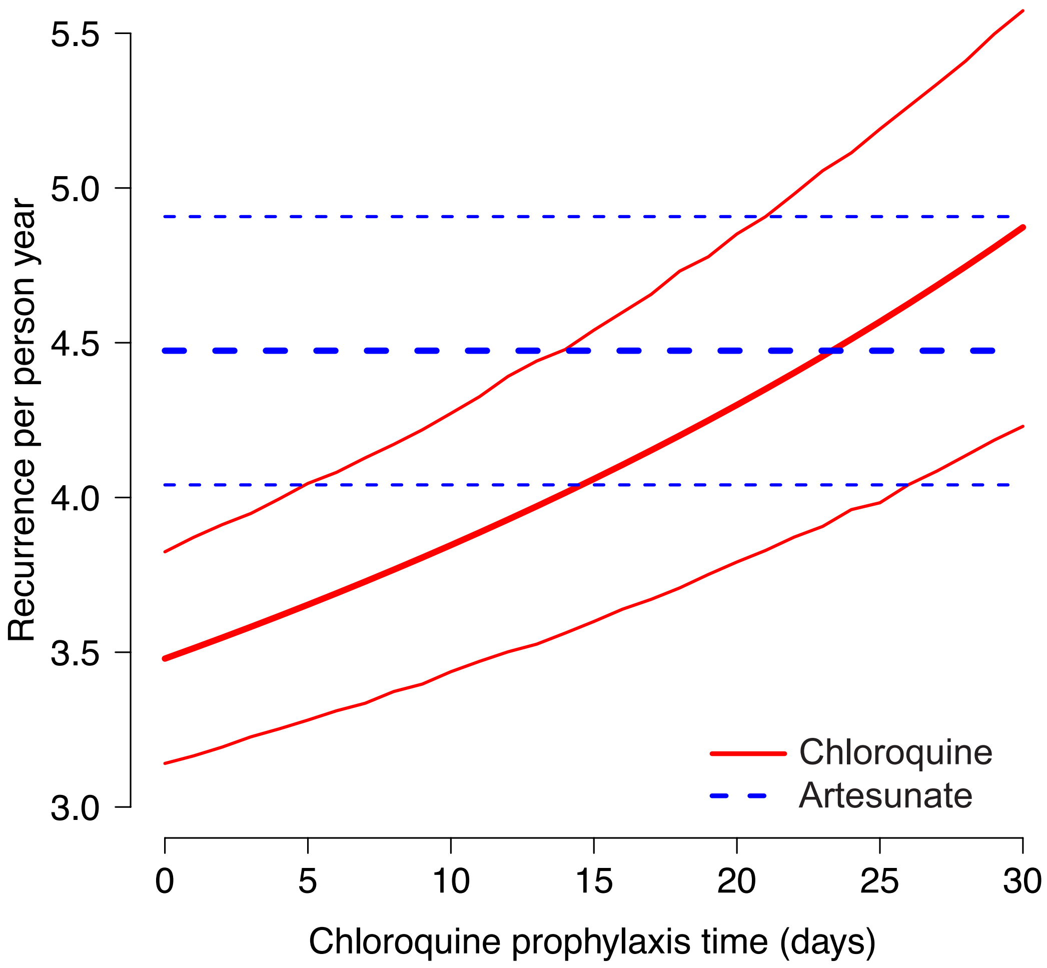

Artesunate is assumed to have no post prophylactic effect and therefore gives the baseline recurrence rate: thick dashed blue line (thin dashed blue lines showing 90% CI). The adjusted annual recurrence rate for chloroquine as a function of the average post treatment prophylactic effect is shown by the thick red line (thin red lines showing 90% CI). A reasonable estimate of the chloroquine post prophylactic effect is ~21 days.

Supplementary Figure 4. Mean absolute hematocrit values up to 5 months after enrolment in patients with a pre-treatment hematocrit ≤ 30% without accounting for recurrences

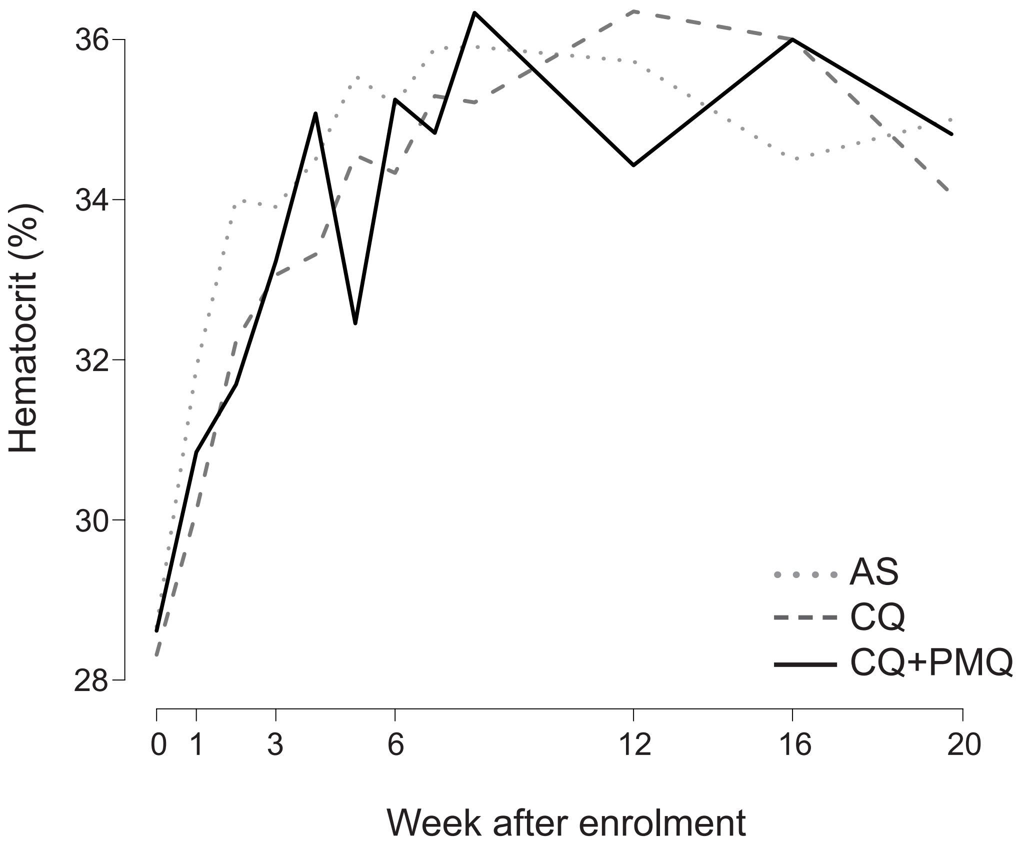

There were 43 patients who were anemic at enrolment. For this analysis anemia was defined as a hematocrit ≤ 30%). At day 6, the mean hematocrit was increased. This was in contrast to the overall hematocrit analysis where the mean hematocrit was at its nadir at day 6. Abbreviations: AS, artesunate; CQ, chloroquine; PMQ, primaquine

Supplementary Figure 5. Median fractional hematocrit recovery at each recurrence

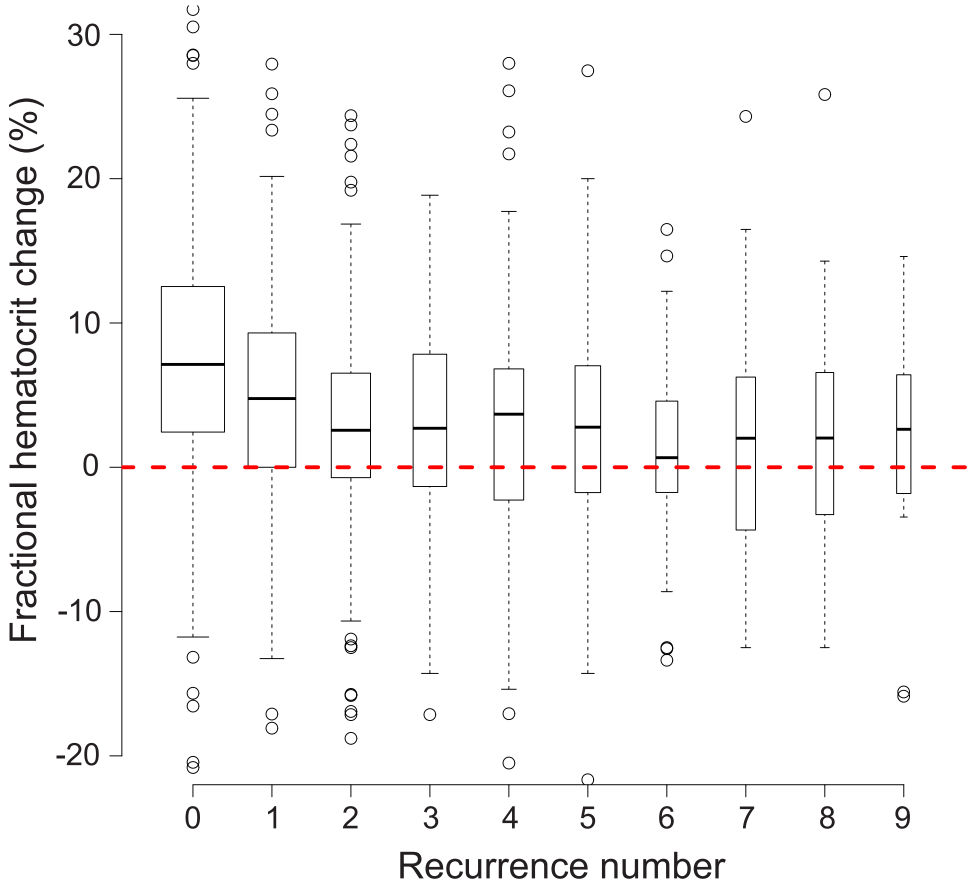

The width of the boxplots is proportional to the square root of the number of individuals in each group. Fractional hematocrit change is calculated by the equation: (steady state hematocrit – day 6 hematocrit) / steady state hematocrit. Steady state hematocrit is the mean of all hematocrit levels taken after day 42 provided that there has been no interim recurrence.

Supplementary Figure 6. The effect of recurrence on the mean absolute hematocrit after the initial *Plasmodium vivax* infection

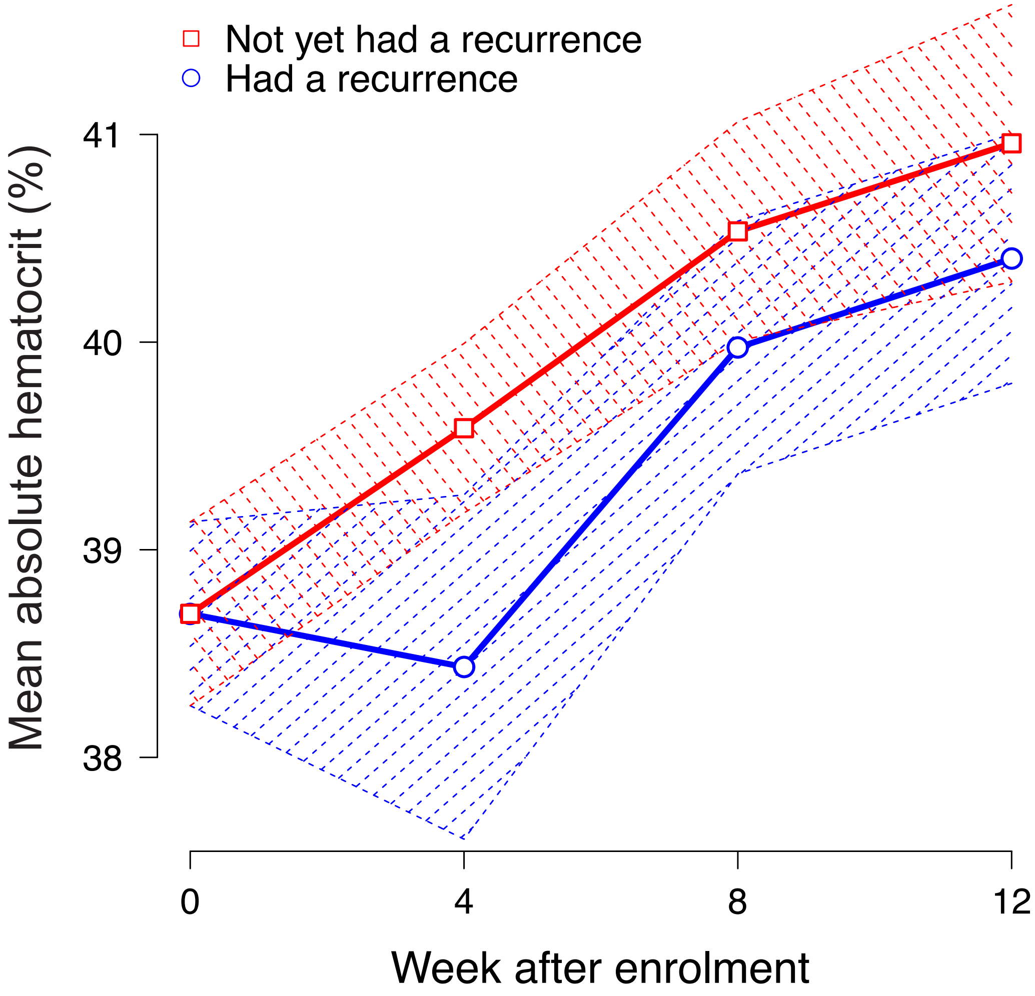

All treatment arms included, data excludes G6PD heterozygous females to remove the confounding of hemolysis caused by primaquine. The shaded areas represent the 95% confidence interval of each mean estimate.
